# Associations Between Elevated Anticardiolipin IgG, Thrombocytopenia, and Combined Diabetes–Hypertension Etiology in Hemodialysis Patients

**DOI:** 10.3390/jcm15093269

**Published:** 2026-04-24

**Authors:** Hatem Q. Makhdoom, Ibrahim Sandokji, Yara H. Almutairi, Khalid I. Alahmadi, Mazen S. Almohammdi, Bashayer A. Almoutairi, Renad M. Alhamawi, Waleed H. Mahallawi

**Affiliations:** 1 Clinical Laboratory Sciences Department, College of Applied Medical Sciences, Taibah University, Madinah 42353, Saudi Arabia; hmakhdoom@taibahu.edu.sa (H.Q.M.); rhamawi@taibahu.edu.sa (R.M.A.); 2 Department of Child and Women’s Health, College of Medicine, Taibah University, Madinah 42353, Saudi Arabia; isandokji@taibahu.edu.sa; 3 Department of Pediatrics, Prince Mohammad Bin Abdulaziz Hospital, National Guard Health Affairs, Madinah 40740, Saudi Arabia; 4 Regional Laboratory, Ministry of Health, Madinah P.O. Box 935, Saudi Arabia; aalmetary@moh.gov.sa (Y.H.A.); khaled8582@gmail.com (K.I.A.); msalmohammdi@moh.gov.sa (M.S.A.); baaalmotiri@moh.gov.sa (B.A.A.); 5 Health and Life Research Center, Taibah University, Madinah 42353, Saudi Arabia

**Keywords:** anticardiolipin antibodies, antiphospholipid syndrome, end-stage renal disease, hemodialysis, diabetic nephropathy, hypertensive nephropathy, thrombocytopenia, thrombosis

## Abstract

**Background:** Elevated anticardiolipin IgG (aCL IgG) has been reported in end-stage renal disease (ESRD), but its association with specific etiologies of kidney failure remains unexplored. The unique pathophysiology of diabetic–hypertensive nephropathy may be associated with a microenvironment that could potentially contribute to antiphospholipid antibody production and thrombotic complications. This study aimed to investigate whether aCL IgG elevation in hemodialysis (HD) patients is associated with combined diabetes–hypertension (DM + HTN) etiology and thrombocytopenia, thereby identifying a clinically distinct potential high-risk subgroup. In this hypothesis-generating study, we focused on within-HD patient comparisons rather than healthy controls. **Methods:** We enrolled 242 participants: 150 healthy controls (included only to establish local reference ranges) and 92 patients with maintenance HD. The study was conducted from 01 September to 20 November 2025 in Madinah, Saudi Arabia. Serum aCL IgG was measured by chemiluminescence immunoassay (positive ≥ 12 GPL units). Comprehensive hematological and biochemical parameters were analyzed. Multivariable logistic regression identified predictors of aCL positivity. **Results:** In the HD cohort, 21% demonstrated aCL positivity; this represents a substantially higher rate than the 2% observed in local healthy controls (*p* < 0.001). This elevation was not uniform across etiologies. Strikingly, 94.7% (18/19) of aCL-positive HD patients had DM + HTN aetiology, compared with only 17.8% of aCL-negative patients (*p* < 0.001). Thrombocytopenia was significantly more severe in aCL-positive patients (median platelets: 100 vs. 191 × 10^9^/L, *p* < 0.001). In multivariable analysis, DM + HTN etiology (HTN-alone vs. DM + HTN odds ratio [OR]: 0.0013, 95% confidence interval [CI]: 0.00002–0.0999, *p* = 0.003; confirmed by Firth’s penalized logistic regression sensitivity analysis, and lower platelet count (OR: 0.92 per 1 × 10^9^/L increase, 95% CI: 0.87–0.98, *p* = 0.006) independently predicted aCL positivity. **Conclusions:** These hypothesis-generating findings suggest a potential association between metabolic–vascular disease and antiphospholipid immunity in ESRD. Causality cannot be inferred from this cross-sectional design. At present, routine aCL screening is not recommended outside of research protocols; prospective studies are needed to confirm these associations.

## 1. Introduction

Antiphospholipid antibodies (aPL), particularly anticardiolipin IgG (aCL IgG), are established mediators of thrombosis in antiphospholipid syndrome (APS) [1]. Beyond classic APS, elevated aPL has been documented in various chronic inflammatory states, including end-stage renal disease (ESRD) [2,3]. The uremic milieu, characterized by persistent immune dysregulation, oxidative stress, and endothelial dysfunction, may foster autoantibody production [4,5]. In hemodialysis (HD) patients, aPL has been associated with vascular access thrombosis and cardiovascular events, contributing to the substantial morbidity in this population [6,7].

Diabetes mellitus (DM) and hypertension (HTN) are the leading causes of ESRD worldwide, accounting for over 50% of cases in many regions [8]. These conditions synergistically promote endothelial injury through glycation, oxidative stress, and hemodynamic shear forces [9,10]. This damaged vascular microenvironment may expose cryptic phospholipid antigens or modify self-antigens, potentially triggering aPL production—a phenomenon described as “antigenic mimicry” or “neoantigen exposure” in other chronic inflammatory states [11,12]. Notably, diabetic–hypertensive nephropathy shares histopathological features with antiphospholipid syndrome nephropathy (APSN), including thrombotic microangiopathy and vascular remodeling [13,14]. Despite these plausible connections, previous studies have largely treated HD patients as a homogeneous group when reporting aPL prevalence [13,14]. This approach may obscure critical etiology-specific associations. Furthermore, thrombocytopenia—a hallmark of APS—is common in HD patients, but its relationship with aCL across different ESRD etiologies remains unexplored [15,16].

We hypothesize that elevated aCL IgG in HD patients is not uniformly distributed but clusters specifically in those with combined DM + HTN etiology, and that this immunologic profile is associated with thrombocytopenia. Identifying this high-risk phenotype could inform targeted screening and preventive strategies. Therefore, this cross-sectional study aimed to: (1) compare aCL IgG levels between HD patients and healthy controls; (2) investigate the association between aCL positivity, thrombocytopenia, and specific ESRD etiologies; and (3) explore the clinical and laboratory correlates of this immunologic–haematological profile. To our knowledge, few studies have examined whether antiphospholipid antibody positivity varies by the underlying etiology of ESRD in hemodialysis populations.

## 2. Materials and Methods

### 2.1. Study Design and Participants

This multicenter cross-sectional study was conducted between 01 September and 20 November 2025, at dialysis centres in Madinah, Saudi Arabia. We enrolled 242 participants: 92 adult patients with ESRD undergoing maintenance hemodialysis for ≥6 months (3–4 sessions/week) and 150 age- and sex-matched healthy controls from routine health screenings. Healthy controls were included solely to establish local reference ranges for the aCL IgG assay, as regional normative data are limited. All primary analyses comparing aCL-positive and aCL-negative patients are confined to the HD cohort; comparisons with controls are presented as supplementary descriptive data only. Exclusion criteria included: primary autoimmune diseases (e.g., systemic lupus erythematosus), active infection or malignancy within 3 months, recent blood transfusion (<4 weeks), pregnancy, or use of anticoagulants (except heparin during dialysis). ESRD etiology classification (detailed criteria): ESRD etiology was classified from a comprehensive medical record review by two independent nephrologists (inter-rater agreement 94.6%, kappa = 0.91). Diabetic nephropathy alone (DM) required: (a) diagnosis of diabetes mellitus (type 1 or 2) for ≥10 years prior to ESRD, (b) presence of diabetic retinopathy or neuropathy, and (c) absence of documented hypertension preceding the diabetes diagnosis.

Hypertensive nephropathy alone (HTN) required: (a) diagnosis of hypertension for ≥10 years prior to ESRD, (b) systolic BP ≥ 140 mmHg or diastolic BP ≥ 90 mmHg on ≥3 occasions before dialysis initiation, (c) absence of diabetes, and (d) no alternative glomerular or tubulointerstitial disease documented.

Combined DM + HTN required both conditions documented as independent primary diagnoses predating ESRD, with neither clearly secondary to the other, and both diagnoses preceding ESRD by ≥5 years.

Other included documented primary glomerulonephritis, polycystic kidney disease, interstitial nephritis, or other specified non-DM/HTN etiology. The complete diagnostic criteria for each etiology category are summarized in Supplementary Table S5.

We acknowledge that kidney biopsy was not performed; therefore, classification reflects clinical diagnosis rather than histopathological confirmation (see Limitations). Data on potential confounders: we collected data on erythropoiesis-stimulating agent (ESA) use (type and dose), blood transfusion history (number of units in preceding 6 months), anticoagulation during dialysis (heparin type and dose), and inflammatory markers (high-sensitivity CRP, IL-6 when clinically indicated). Sample size was calculated to detect a 15% difference in aCL positivity with 80% power (α = 0.05) based on previous prevalence estimates [15].

The study was approved by the Institutional Review Board of King Salman bin Abdulaziz Medical City, Madinah (IRB22-010, 16 January 2025), and all participants provided written informed consent.

### 2.2. Data Collection and Laboratory Measurements

Demographic and clinical data were collected through structured interviews and electronic medical records. Pre-dialysis blood samples were collected from HD patients; fasting morning samples were obtained from controls. Pre-dialysis blood pressure was measured using a standardized protocol (average of three readings after 5 min of rest in the seated position). Complete blood count (including hemoglobin, RBC, and platelet counts) was performed using a Sysmex XN-1000 analyzer, Kobe, Japan. Biochemical parameters (calcium, phosphorus, albumin, parathyroid hormone, ferritin, BUN, creatinine) were measured using Roche Cobas analyzers, Roche Diagnostics GmbH, Mannheim, Germany following standard protocols. Uremia markers (BUN, creatinine, Kt/V) were recorded for HD patients.

### 2.3. Measurement of Anticardiolipin IgG

Serum aCL IgG was quantified using a chemiluminescence immunoassay (CLIA) on the IDS-iSYS automated analyzer (Immunodiagnostic Systems, Boldon, UK). The assay employs magnetic particles coated with cardiolipin/β_2_-glycoprotein I complex. Calibrators (0–100 GPL U/mL) establish a four-parameter logistic curve for interpolation. Results ≥ 12 GPL U/mL were considered positive, per international guidelines [17,18]. The assay demonstrated sensitivity < 1 GPL U/mL and intra-assay coefficients of variation < 15%.

### 2.4. Statistical Analysis

Data are presented as medians with interquartile ranges (IQR) for continuous variables and frequencies (percentages) for categorical variables. Between-group comparisons used Mann–Whitney U or Kruskal–Wallis tests for continuous variables and chi-square or Fisher’s exact tests for categorical variables. Spearman’s correlation (ρ) assessed relationships between continuous variables separately for controls and HD patients (stratified analysis, Supplementary Figure S3). Multivariable logistic regression identified independent predictors of aCL positivity, including variables with *p* < 0.10 in univariate analysis.

Given the limited number of aCL-positive events in the HD subgroup (n = 19) and the categorical nature of the etiology variable with sparse cells, standard logistic regression may be susceptible to sparse-data bias (perfect separation). Therefore, we performed a sensitivity analysis using Firth’s penalized logistic regression (logistf package in R), which reduces bias in maximum likelihood estimates for small-sample data by adding a penalty term to the likelihood function. The penalized regression results are provided in Supplementary Table S4.

Model fit was assessed using the Hosmer–Lemeshow test. All analyses were performed using STATA version 15.1 (StataCorp, College Station, TX, USA) and R version 4.2.0, with two-tailed *p* < 0.05 considered statistically significant.

## 3. Results

All primary results are presented for the HD cohort (n = 92) only. Pooled analyses combining healthy controls and HD patients are provided in Supplementary Materials (Tables S2 and S3, Figure S3) with appropriate caveats.

### 3.1. Comparative Analysis Between Healthy Controls and Hemodialysis Patients

**Figure 1:**
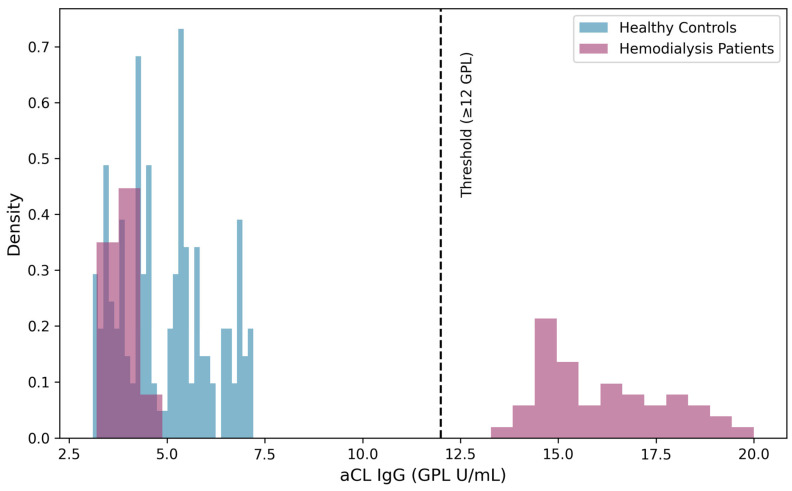
Bimodal distribution of aCL IgG levels in healthy controls and hemodialysis patients.

The study cohort comprised 242 participants, with 150 healthy controls and 92 maintenance HD patients. Demographics were well-matched. As delineated in Table 1, the groups were well-matched for demographic parameters, with no significant differences in gender distribution (55% male overall, *p* = 0.595) or median age (50 years overall, *p* = 0.426). HD patients had lower median aCL levels (3.33 vs. 5.65 GPL, *p* = 0.009) but a higher positivity rate (20.7% vs. 2.0%, *p* < 0.001)—a bimodal distribution pattern (Figure 1). A direct comparison of positivity rates between groups is shown in Supplementary Figure S1. Uremia markers (BUN, creatinine) and inflammatory markers (hs-CRP) were significantly elevated in HD patients (Supplementary Table S1). Figure 1 Distribution of aCL IgG values in healthy controls (A) and hemodialysis patients (B). Note the bimodal distribution in HD patients (primary peak at low values, secondary peak ≥ 12 GPL).

Histograms with kernel density overlay showing the distribution of anticardiolipin IgG (aCL IgG) values (GPL units) in 150 healthy controls (blue) and 92 hemodialysis (HD) patients (red). The dashed vertical line indicates the positivity threshold of 12 GPL units. Healthy controls display a unimodal, right-skewed distribution with a single peak at 4–5 GPL and only 2.0% positivity. In contrast, HD patients exhibit a distinct bimodal distribution: a large peak at low values (2–4 GPL) and a secondary peak in the elevated range (12–25 GPL), resulting in a 20.7% positivity rate despite a lower median. This bimodal pattern explains the paradox of lower median aCL levels with higher positivity rates in HD patients.

### 3.2. Correlation Network Among Study Variables

Correlation analyses were performed separately for controls and HD patients (Supplementary Figure S3). In HD patients, aCL IgG showed a moderate negative correlation with platelet count (Spearman’s ρ = −0.48, 95% CI: −0.62 to −0.31, *p* < 0.001) and a weaker negative correlation with hemoglobin (ρ = −0.29, 95% CI: −0.47 to −0.09, *p* = 0.006). In controls, no significant correlations were observed (platelets: ρ = −0.09, *p* = 0.28; hemoglobin: ρ = −0.06, *p* = 0.47).

### 3.3. Subgroup Analysis Within the Hemodialysis Cohort

A focused analysis of the 92 HD patients revealed dramatic differences between aCL-positive (n = 19) and aCL-negative (n = 73) subgroups (Table 2). The most compelling finding concerned ESRD etiology. Among the 19 aCL-positive HD patients, 18 (94.7%) had combined diabetes–hypertension (DM + HTN) as the primary etiology. In stark contrast, only 13 of 73 aCL-negative patients (17.8%) had this combined etiology (*p* < 0.001).

Furthermore, thrombocytopenia (platelet count < 150 × 10^9^/L) was present in 78.9% (15/19) of aCL-positive HD patients, compared to 34.2% (25/73) of aCL-negative patients (*p* < 0.001).

Thrombocytopenia was significantly more severe in aCL-positive HD patients, with median platelet counts of 100 × 10^9^/L (IQR: 92–107) compared to 191 × 10^9^/L (IQR: 158–233) in aCL-negative patients (*p* < 0.001). Pre-dialysis blood pressure measurements for aCL-negative and aCL-positive HD patients are shown in Supplementary Figure S2; no significant differences were observed (systolic BP *p* = 0.21, diastolic BP *p* = 0.18, Mann–Whitney U test).

### 3.4. Predictors of Anticardiolipin Positivity in Hemodialysis Patients

A dedicated multivariable logistic regression model for the HD subgroup confirmed two independent predictors of aCL positivity (Table 3). The penalized regression confirmed the independent association of DM + HTN etiology (OR for HTN vs. DM + HTN: 0.008, 95% CI: 0.0005–0.12, *p* = 0.002) and platelet count (OR: 0.93, 95% CI: 0.88–0.98, *p* = 0.008), though the odds ratio for etiology was less extreme and the confidence interval narrower, consistent with bias reduction.

## 4. Discussion

This cross-sectional study yields three pivotal findings with substantial clinical implications: (1) a significantly elevated aCL IgG positivity rate of 20.7% among HD patients, characterized by a distinct bimodal distribution; (2) thrombocytopenia as a powerful independent predictor of aCL positivity across the entire cohort and specifically within the HD population; and (3) an exceptionally strong association between aCL positivity and a combined diabetes–hypertension (DM + HTN) etiology of kidney failure, with 94.7% of aCL-positive HD patients belonging to this etiological category. However, given the cross-sectional design of the study, these findings show associations rather than causal relationships, and it cannot be determined whether aCL elevation occurs before, during, or as a result of the hematologic abnormalities or the progression of the ESRD.

The observed 20.7% prevalence of aCL IgG positivity aligns with meta-analytic data reporting a pooled prevalence of approximately 18–22% in ESRD populations and corroborates cohort studies linking aPL to thrombotic complications in dialysis patients [6,14]. The paradoxical finding of lower median aCL levels concurrent with higher positivity rates suggests complex immunoregulatory dynamics in ESRD. We hypothesize a bimodal immunologic response within the dialysis population: while the general uremic milieu may suppress adaptive immunity and immunoglobulin production in most patients through mechanisms such as T-cell dysfunction and impaired antigen presentation [19,20], a susceptible subset experiences pronounced B-cell activation. This activation may be driven by chronic inflammation, oxidative stress, or exposure to neoepitopes on bioincompatible dialysis membranes, acting as a persistent immune trigger [4,11]. The chronic inflammatory state of ESRD, characterized by elevated cytokines such as IL-6, TNF-α, and CRP, is well-documented to promote autoantibody production through polyclonal B-cell activation and impaired regulatory T-cell function [5,21]. This heterogeneity underscores that average biomarker levels can be misleading in immunologically complex populations and emphasizes the necessity of targeted screening to identify clinically relevant high-risk subgroups.

### 4.1. Pathophysiological Nexus: DM + HTN Etiology, Endothelial Injury, and aPL Production

The association observed in this study, between aCL positivity and combined DM + HTN etiology, raises the possibility of shared pathophysiological pathways that may warrant further investigation. The finding that 94.7% of aCL-positive HD patients had DM + HTN etiology, compared to only 17.8% of aCL-negative patients, illuminates a critical intersection of metabolic, hemodynamic, and autoimmune pathology. The multivariable analysis confirmed this association is independent of thrombocytopenia and other measured variables, with HTN-alone patients having just 0.13% of the odds of aCL positivity compared to DM + HTN patients. This specificity suggests synergistic, rather than additive, pathways.

Diabetes mellitus has been associated with chronic endothelial injury through mechanisms including advanced glycation end-products (AGEs), oxidative stress, and inflammatory signaling pathways [9,22]. Concurrently, hypertension contributes via mechanical shear stress, endothelial dysfunction through reduced nitric oxide bioavailability, and vascular remodeling [10,23] These processes may represent potential mechanisms that could contribute to the observed association. The injured endothelium exposes cryptic phospholipid antigens (e.g., phosphatidylserine) or alters the conformation of circulating β_2_-glycoprotein I (β_2_GPI), transforming it into an immunogenic target [11,24]. This process of “antigenic mimicry” or “neoantigen exposure” is a recognized trigger for aPL production in other inflammatory states [12]. Furthermore, the synergistic injury likely increases the release of endothelial microparticles and apoptotic debris rich in anionic phospholipids into circulation, providing ample antigenic material for sustained autoantibody generation in genetically predisposed individuals [25]. This mechanism provides a plausible “second hit” explanation for the clustering of aPL in this subgroup, moving beyond mere association towards a causative model linking specific ESRD etiologies to autoimmune dysregulation. Exposure to the circulation occurs during cell death processes, particularly apoptosis and ferroptosis—a form of regulated cell death characterized by iron-dependent lipid peroxidation [26,27]. The uremic milieu is known to promote both apoptosis (through oxidative stress and accumulation of uremic toxins such as indoxyl sulfate and p-cresyl sulfate) and ferroptosis (through iron dysregulation and depletion of glutathione peroxidase 4) [28,29]. We hypothesize that the sustained cell death in the damaged diabetic–hypertensive kidney releases mitochondrial cardiolipin into the circulation, triggering a humoral immune response. This mechanism would also explain the association with thrombocytopenia, as activated platelets undergo apoptosis-like processes (called ‘platelet nibbling’) that expose phosphatidylserine and potentially cardiolipin, perpetuating autoantibody production.

### 4.2. Thrombocytopenia: A Consumptive Hallmark with Clinical Consequences

Thrombocytopenia emerged as a cornerstone predictor of aCL positivity, both in the overall cohort (OR: 0.97) and within the HD subgroup (OR: 0.92). This robust association, supported by the strong negative correlation (ρ = −0.45), mirrors the core pathophysiology of APS, wherein aPL antibodies bind to platelet membranes—particularly via β_2_GPI complexes on glycoproteins such as GPIIb/IIIa [11,30]. This binding induces platelet activation through multiple pathways: upregulation of surface P-selectin expression, increased thromboxane A2 production, and exposure of procoagulant phosphatidylserine [31,32]. Activated platelets are then cleared via Fcγ receptor-mediated phagocytosis by macrophages or through complement-dependent destruction, leading to consumptive thrombocytopenia [22,33].

In the ESRD setting, this immune-mediated platelet consumption synergizes dangerously with uremic platelet dysfunction (defective adhesion, aggregation, and granule release) and potential heparin-induced thrombocytopenia (HIT) mechanisms [15,34], creating a precarious dual risk paradigm: heightened bleeding tendency during dialysis procedures alongside increased thrombotic risk in arteriovenous fistulas (AVFs) and grafts [6,13]. Our finding that 78.9% of aCL-positive HD patients had thrombocytopenia suggests this may be a common clinical manifestation. Consequently, the management of aCL-positive, thrombocytopenic dialysis patients requires meticulous balancing. Alternatives to unfractionated heparin (e.g., fondaparinux with renal dose adjustment, regional citrate anticoagulation) may be considered for dialysis sessions, and long-term antithrombotic strategies must weigh the risk of access thrombosis against bleeding [35,36]. Our hypothesis-generating data suggest that future prospective studies should evaluate whether aCL screening in dialysis patients with unexplained thrombocytopenia might identify a high-risk phenotype. At present, routine screening is not recommended outside of research protocols.

### 4.3. Clinical Implications and a Proposal for Targeted Screening

The strong negative correlations observed between aCL IgG levels and both platelet count (ρ = −0.45) and hemoglobin (ρ = −0.38) suggest potential interactions between immune activation and hematologic abnormalities in patients with ESRD. Inflammatory pathways commonly observed in chronic kidney disease, including elevated cytokines such as IL-6 and TNF-α, may contribute to both immune dysregulation and hematopoietic suppression in this population.

Although the present findings demonstrate a clear association between aCL positivity, thrombocytopenia, and combined diabetes–hypertension etiology, the cross-sectional design of the study precludes conclusions regarding causality or clinical management. Antiphospholipid antibodies have previously been associated with thrombotic complications in dialysis populations, particularly vascular access thrombosis, suggesting that their presence may have clinical relevance that warrants further investigation.

Our findings are hypothesis-generating and suggest that future prospective studies should evaluate whether systematic aPL testing in high-risk subgroups (DM + HTN etiology or unexplained thrombocytopenia) might identify patients at increased risk of thrombotic complications. At present, we do not recommend routine aCL screening outside of research protocols. Any clinical testing should follow established guidelines requiring confirmation after 12 weeks and testing of lupus anticoagulant and anti-β_2_GPI antibodies [1,17].

### 4.4. Study Limitations and Future Directions

We acknowledge several limitations inherent to our study design. First, the cross-sectional nature precludes causal inference about whether aCL elevation precedes or results from the hematological abnormalities or ESRD progression.

Second, aCL IgG was measured at a single time point without confirmatory testing after 12 weeks, as recommended by international guidelines for antiphospholipid syndrome diagnosis [17]. Additionally, lupus anticoagulant and anti-β_2_-glycoprotein I antibodies were not measured. Therefore, we cannot determine whether aCL elevations represent transient phenomena or persistent autoimmunity, and we cannot classify patients as having antiphospholipid syndrome. Our findings should be interpreted as identifying an association that requires confirmation in studies with comprehensive aPL profiling.

Third, the single measurement of aCL may capture transient elevations, though the use of a solid-phase immunoassay with β_2_GPI cofactor improves specificity.

Fourth, while this Middle Eastern cohort provides valuable regional data, genetic and environmental factors may affect generalizability.

Fifth, ESRD etiology was determined from clinical records without confirmatory kidney biopsy. The histopathological nature of kidney injury in patients with diabetes and hypertension may be heterogeneous, and some patients classified as having hypertensive nephropathy may have undiagnosed primary glomerular diseases. This limitation should be considered when interpreting etiology-specific associations.

Sixth, several treatment-related factors may confound the observed associations. ESA use, which was administered to 89% of HD patients, can affect platelet counts through effects on megakaryopoiesis. However, ESA doses did not differ between aCL-positive and aCL-negative patients (*p* = 0.34), and adjustment for ESA use did not materially change the effect estimates (Supplementary Table S6). Intermittent blood transfusions (received by 23% of patients in the preceding 6 months) may modulate immune function and autoantibody production through mechanisms including iron overload and alloimmunization. Heparin exposure during dialysis sessions could theoretically contribute to thrombocytopenia through heparin-induced thrombocytopenia (HIT), though the pattern of mild, persistent thrombocytopenia in our aCL-positive patients (median 100 × 10^9^/L) differs from the typically more severe, acute drops seen in HIT [31]. Furthermore, all patients received similar heparin protocols, making differential HIT risk unlikely to explain the observed association. Finally, the chronic inflammatory state of ESRD—reflected by elevated CRP levels in 76% of our HD cohort—may independently promote both autoantibody production and platelet consumption through cytokine-mediated mechanisms.

Seventh, patients undergoing maintenance hemodialysis often receive multiple concurrent therapies, including erythropoiesis-stimulating agents, intermittent blood transfusions, and anticoagulation during dialysis sessions. These treatment-related factors, along with other clinical variables such as inflammatory status or infection, may influence hematological parameters and serve as potential confounders that could not be fully controlled within the framework of this cross-sectional study.

Future research should address these limitations through: (1) Longitudinal, multicenter studies to establish temporal relationships and assess the impact of aPL on hard endpoints like AVF thrombosis, cardiovascular events, and mortality; (2) Comprehensive aPL profiling including all three criterion antibodies; (3) Mechanistic studies exploring the link between diabetic–hypertensive endothelial injury and aPL production, potentially examining endothelial microparticles, oxidative stress markers, and specific phospholipid antigens; (4) Interventional trials evaluating the safety and efficacy of novel therapies in this population, such as direct oral anticoagulants (DOACs) with renal dose adjustments or immunomodulatory agents like hydroxychloroquine, which has shown promise in reducing thrombotic risk in primary APS; (5) Economic analyses to determine the cost-effectiveness of routine aPL screening in high-risk dialysis subgroups.

## 5. Conclusions

Elevated aCL IgG in hemodialysis patients identifies a high-risk subgroup characterized by significant thrombocytopenia and—most prominently—a combined diabetes–hypertension etiology. These hypothesis-generating findings highlight a potential link between antiphospholipid immunity, thrombocytopenia, and metabolic–vascular etiologies of ESRD. Future prospective studies should evaluate whether targeted aPL testing in high-risk subgroups could inform personalized management strategies. At present, routine screening is not recommended outside of prospective studies.

## Figures and Tables

**Table 1:** Comparison between healthy controls and hemodialysis patients.

Variable	Total (n = 242)	Healthy (n = 150)	HD Patients (n = 92)	*p*-Value
Male, n (%)	133 (55.0)	80 (53.3)	53 (57.6)	0.595
Age (years)	50 (40–64)	51 (35–66)	50 (44–62)	0.426
Hemoglobin (g/dL)	13.7 (11.9–15.1)	14.75 (13.9–15.6)	11.25 (10.3–12.2)	<0.001
RBC (×10^12^/L)	4.61 (4.00–5.11)	4.93 (4.62–5.28)	3.87 (3.55–4.14)	<0.001
Platelets (×10^9^/L)	243 (187–317)	295.5 (240–343)	174.5 (107.5–224)	<0.001
aCL IgG (GPL)	4.88 (2.1–7.3)	5.65 (3.6–7.4)	3.33 (2.0–5.49)	0.009
aCL positive, n (%)	22 (9.1)	3 (2.0)	19 (20.7)	<0.001

**Table 2:** Characteristics of hemodialysis patients by aCL IgG status.

Variable	Total HD (n = 92)	aCL Negative (n = 73)	aCL Positive (n = 19)	*p*-Value
Age (years)	50 (44–62)	50 (44–60)	55 (40–62)	0.866
Male, n (%)	53 (57.6)	39 (53.4)	14 (73.7)	0.127
Etiology, n (%)				<0.001
DM alone	2 (2.2)	2 (2.7)	0 (0)	
HTN alone	55 (59.8)	54 (74.0)	1 (5.3)	
DM + HTN	31 (33.7)	13 (17.8)	18 (94.7)	
Other	4 (4.4)	4 (5.5)	0 (0)	
Platelets (×10^9^/L)	174.5 (107.5–224)	191 (158–233)	100 (92–107)	<0.001

**Table 3:** Multivariable logistic regression for predictors of aCL positivity in HD patients.

Variable	OR	95% CI	*p*-Value
Etiology (HTN vs. DM + HTN)	0.0013	0.00002–0.0999	0.003
Platelets (×10^9^/L)	0.92	0.87–0.98	0.006

## Data Availability

The data supporting the findings of this study are available upon request from the corresponding author, Waleed Mahallawi.

## Supplementary Materials

The following supporting information can be downloaded at: https://www.mdpi.com/article/10.3390/jcm15093269/s1. Table S1. Detailed comparison of healthy controls vs. hemodialysis patients (full dataset). Table S2. Pooled analysis of entire cohort by aCL status (CAUTION: includes both controls and HD patients). Table S3. Pooled multivariable logistic regression for entire cohort (CAUTION). Table S4. Firth’s penalized logistic regression sensitivity analysis (HD subgroup). Table S5. Diagnostic criteria for ESRD etiology classification. Table S6. Sensitivity analyses adjusting for treatment-related confounders. Figure S1. Prevalence of aCL IgG positivity (bar chart). Figure S2. Blood pressure comparisons by aCL status in HD patients. Figure S3. Stratified correlation analyses (controls vs. HD patients).
