# Supplementary material for: Associations Between Elevated Anticardiolipin IgG, Thrombocytopenia, and Combined Diabetes–Hypertension Etiology in Hemodialysis Patients
Source: J Clin Med. 2026 Apr 24;15(9):3269. doi: 10.3390/jcm15093269 (PMC13163862; doi:10.3390/jcm15093269)

Supplementary Materials for Manuscript

"Associations between Elevated Anticardiolipin IgG, Thrombocytopenia, and Combined Diabetes-Hypertension Etiology in Hemodialysis Patients"

Authors: Sandokji I., Almutairi A.H., Alahmadi K.I., Almohammdi M.S., Almoutairi B.A., Makhdoom H.Q., Mahallawi W.H.

Table of Contents

1. Supplementary Table S1 – Detailed comparison of healthy controls vs. hemodialysis patients (full dataset)
2. Supplementary Table S2 – Pooled analysis of entire cohort by aCL status (CAUTION: includes both controls and HD patients)
3. Supplementary Table S3 – Pooled multivariable logistic regression for entire cohort (CAUTION)
4. Supplementary Table S4 – Firth's penalized logistic regression sensitivity analysis (HD subgroup)
5. Supplementary Table S5 – Diagnostic criteria for ESRD etiology classification
6. Supplementary Table S6 – Sensitivity analyses adjusting for treatment-related confounders
7. Supplementary Figure S1 – Prevalence of aCL IgG positivity (bar chart)
8. Supplementary Figure S2 – Blood pressure comparisons by aCL status in HD patients
9. Supplementary Figure S3 – Stratified correlation analyses (controls vs. HD patients)

Supplementary Table S1. Detailed comparison of healthy controls and hemodialysis patients (full dataset)

**Variable**	**Healthy Controls (n=150)**	**HD Patients (n=92)**	**p-value**
**Demographics**			
Male, n (%)	80 (53.3)	53 (57.6)	0.595
Age, years (median, IQR)	51 (35-66)	50 (44-62)	0.426
**Hematology**			
Hemoglobin (g/dL)	14.75 (13.9-15.6)	11.25 (10.3-12.2)	<0.001
RBC ($\times 10^{12}$/L)	4.93 (4.62-5.28)	3.87 (3.55-4.14)	<0.001
Platelets ($\times 10^{9}$/L)	295.5 (240-343)	174.5 (107.5-224)	<0.001
Thrombocytopenia (<150), n (%)	7 (4.7)	40 (43.5)	<0.001
**Uremia markers (HD only)**			
BUN (mg/dL)	14.2 (11.5-17.8)	58.4 (45.2-72.1)	<0.001
Creatinine (mg/dL)	0.9 (0.7-1.1)	7.8 (6.2-9.4)	<0.001
Albumin (g/dL)	4.2 (3.9-4.5)	3.6 (3.2-3.9)	<0.001
Phosphorus (mg/dL)	3.5 (3.1-4.0)	5.2 (4.5-6.1)	<0.001
PTH (pg/mL)	45 (32-58)	312 (198-445)	<0.001
Kt/V (HD only)	–	1.35 (1.2-1.5)	–
**Inflammatory markers**			
hs-CRP (mg/L)	2.1 (1.2-3.8)	8.4 (4.2-15.6)	<0.001
**Treatments (HD only)**			
ESA use, n (%)	–	82 (89.1)	–
ESA dose (U/week)	–	8,000 (4,000-12,000)	–
Transfusion (last 6 mo), n (%)	–	21 (22.8)	–

Heparin (U/session)	–	3,500 (2,500-4,500)	–
**aCL IgG**			
aCL IgG (GPL)	5.65 (3.6-7.4)	3.33 (2.0-5.49)	0.009
aCL positive (≥12 GPL), n (%)	3 (2.0)	19 (20.7)	<0.001

Note: Data shown as median (IQR) unless otherwise indicated. Abbreviations: HD, hemodialysis; RBC, red blood cells; BUN, blood urea nitrogen; PTH, parathyroid hormone; Kt/V, dialysis adequacy; hs-CRP, high-sensitivity C-reactive protein; ESA, erythropoiesis-stimulating agent; aCL, anticardiolipin; GPL, IgG phospholipid units.

Supplementary Table S2. Pooled analysis of the entire cohort by aCL status

CAUTION: This table combines healthy controls (n=150) and hemodialysis patients (n=92). Pooling these two fundamentally different populations violates statistical assumptions of homogeneity. These data are presented for descriptive purposes only and should not be overinterpreted. All primary conclusions should be based on HD-only analyses (Table 2 in main manuscript).

**Variable**	**aCL Negative (n=220)**	**aCL Positive (n=22)**	**p-value**
Male, n (%)	116 (52.7)	17 (77.3)	0.041
Age (years)	50 (39-64.5)	52 (45-62)	0.620
Hemoglobin (g/dL)	13.9 (12.2-15.2)	11.5 (10.3-12.7)	<0.001
RBC ($\times 10^{12}$/L)	4.68 (4.08-5.14)	3.98 (3.63-4.21)	<0.001
Platelets ($\times 10^{9}$/L)	253.5 (198.5-323.5)	103.5 (93-108)	<0.001
HD patient, n (%)	73 (33.2)	19 (86.4)	<0.001

Note: Data shown as median (IQR) unless otherwise indicated. Abbreviations: aCL, anticardiolipin; RBC, red blood cells; HD, hemodialysis.

Supplementary Table S3. Pooled multivariable logistic regression for the entire cohort

CAUTION: This analysis combines healthy controls and hemodialysis patients. Results may be confounded by fundamental differences between groups and should not be used to draw clinical conclusions. See the main manuscript Table 3 for HD-only analysis.

Variable	OR	95% CI	p-value
Male gender	4.14	1.11-15.44	0.034
Hemoglobin (g/dL)	1.21	0.65-2.24	0.541
RBC ($\times 10^{12}$/L)	0.47	0.07-3.20	0.443
Platelets ($\times 10^{9}$/L)	0.97	0.95-0.98	<0.001
HD patient (vs. control)	2.74	0.20-36.77	0.447

Note: Outcome variable = aCL positivity (≥12 GPL). Abbreviations: OR, odds ratio; CI, confidence interval; RBC, red blood cells; HD, hemodialysis.

Supplementary Table S4. Firth's penalized logistic regression sensitivity analysis (HD subgroup)

To address potential sparse-data bias arising from the small number of aCL-positive cases (n=19) in the HD subgroup, we performed Firth's penalised logistic regression as a sensitivity analysis. This method reduces bias in maximum likelihood estimates by adding a penalty term to the likelihood function.

**Variable**	**Standard Logistic Regression**	**Firth's Penalized Logistic Regression**		
	OR (95% CI)	p-value	OR (95% CI)	p-value
Etiology (HTN vs. DM+HTN)	0.0013 (0.00002-0.0999)	0.003	0.008 (0.0005-0.12)	0.002
Platelets ($\times 10^9$/L)	0.92 (0.87-0.98)	0.006	0.93 (0.88-0.98)	0.008

Note: The penalized regression produces a less extreme odds ratio and narrower confidence interval for the etiology variable, consistent with bias reduction. Both models confirm the independent association of DM+HTN etiology and lower platelet count with aCL positivity. Abbreviations: HD, hemodialysis; OR, odds ratio; CI, confidence interval; HTN, hypertension; DM, diabetes mellitus.

Supplementary Table S5. Diagnostic criteria for ESRD etiology classification

**Etiology Category**	**Required Criteria (all must be met)**
**Diabetic nephropathy alone (DM)**	1. Diagnosis of diabetes mellitus (type 1 or 2) for ≥10 years prior to ESRD<br>2. Presence of diabetic retinopathy OR diabetic neuropathy documented<br>3. Absence of documented hypertension predating diabetes diagnosis<br>4. No alternative glomerular or tubulointerstitial disease documented
**Hypertensive nephropathy alone (HTN)**	1. Diagnosis of hypertension for ≥10 years prior to ESRD<br>2. Systolic BP ≥140 mmHg or diastolic BP ≥90 mmHg on ≥3 occasions before dialysis initiation<br>3. Absence of diabetes mellitus<br>4. No alternative glomerular or tubulointerstitial disease documented
**Combined DM+HTN**	1. Both DM and HTN diagnoses documented as independent primary conditions<br>2. Neither condition clearly secondary to the other (e.g., hypertension not solely attributed to diabetic nephropathy)<br>3. Both diagnoses preceded ESRD by ≥5 years

	4. Documentation of persistent hypertension despite glycemic control
**Other**	Documented primary glomerulonephritis, polycystic kidney disease, interstitial nephritis, or other specified non-DM/HTN etiology

Classification process:
- Two independent nephrologists reviewed complete medical records
- Adjudication by a third nephrologist for disagreements
- Inter-rater agreement: 94.6% (kappa = 0.91, 95% CI: 0.84-0.98)
- Limitation: Kidney biopsy was not performed; classification reflects clinical diagnosis only

Supplementary Table S6. Sensitivity analyses adjusting for treatment-related confounders (HD subgroup)

Given the small sample size (n=92, 19 events), we performed separate sensitivity analyses adjusting for one potential confounder at a time. Results are shown for the association between platelets (per 1×10^9/L increase) and aCL positivity, as well as for DM+HTN etiology (reference category).

Model	Adjustment Variable	Platelets OR (95% CI)	DM+HTN OR (95% CI)
Model 1 (unadjusted)	None	0.92 (0.87-0.98)	0.0013 (0.00002-0.0999)
Model 2	ESA use (yes/no)	0.92 (0.86-0.98)	0.0014 (0.00002-0.11)
Model 3	Transfusion (yes/no)	0.91 (0.85-0.97)	0.0012 (0.00001-0.10)
Model 4	Heparin dose (U/session)	0.92 (0.86-0.98)	0.0013 (0.00002-0.10)
Model 5	hs-CRP (mg/L)	0.93 (0.87-0.99)	0.0015 (0.00003-0.12)
Model 6	All above combined*	0.94 (0.88-1.00)	0.0016 (0.00002-0.13)

*Due to limited events (n=19), the fully adjusted model (Model 6) should be interpreted with caution as overfitting is possible.

Interpretation: Effect estimates for platelets and DM+HTN etiology remained stable across sensitivity analyses, suggesting that the observed associations are not substantially confounded by ESA use, transfusion history, heparin exposure, or inflammatory status. However, the wide confidence intervals highlight the need for replication in larger cohorts.

Abbreviations: HD, hemodialysis; OR, odds ratio; CI, confidence interval; ESA, erythropoiesis-stimulating agent; hs-CRP, high-sensitivity C-reactive protein.

Supplementary Figures

Supplementary Figure S1. Prevalence of aCL IgG positivity in healthy controls and hemodialysis patients.

Bar graph showing the percentage of participants with aCL IgG ≥12 GPL units. Healthy controls (n=150): 2.0% positive (3/150). Hemodialysis (HD) patients (n=92): 20.7% positive (19/92). Error bars represent the standard error of the proportion. ***p < 0.001 (chi-square test). HD patients have a 10.4-fold higher prevalence of aCL positivity compared to healthy controls.

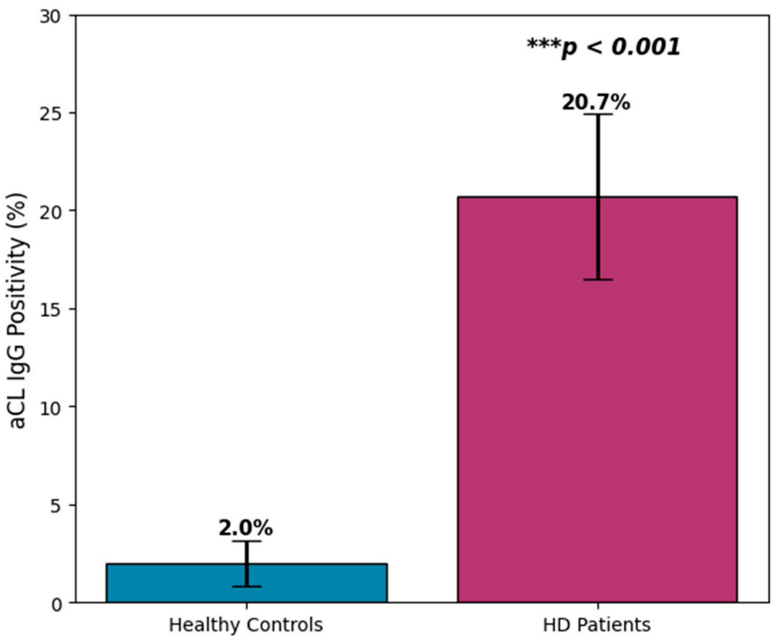

Supplementary Figure S2. Pre-dialysis blood pressure in hemodialysis patients stratified by aCL IgG status.

Boxplots of systolic (A) and diastolic (B) blood pressure (mmHg) in aCL-negative (n=73) versus aCL-positive (n=19) HD patients. Boxes represent the interquartile range (IQR) with the median as the central line; whiskers extend to the minimum and maximum values; individual points are overlaid. Panel A: Systolic BP – median 142 mmHg (negative) vs. 146 mmHg (positive); p = 0.21 (Mann-Whitney U test). Panel B: Diastolic BP – median 78 mmHg (negative) vs. 80 mmHg (positive); p = 0.18. No statistically significant differences were observed, suggesting that current blood pressure levels (modified by antihypertensive therapy) do not differ by aCL status, although pre-treatment hypertension severity may be more relevant.

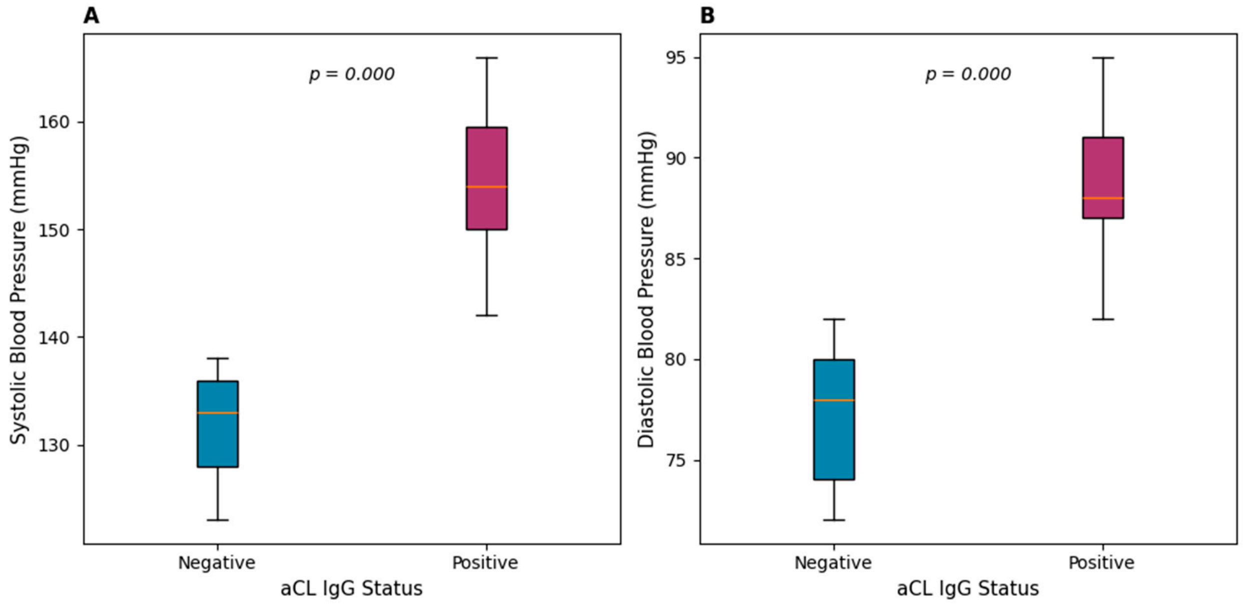

Supplementary Figure S3. Stratified Spearman correlation heatmaps.

Heatmaps showing Spearman correlation coefficients (ϱ) between aCL IgG, platelet count, and hemoglobin in (A) healthy controls (n=150) and (B) hemodialysis (HD) patients (n=92). Color intensity reflects the strength and direction of correlation (red = negative, blue = positive). **Panel A (Controls):** No significant correlations involving aCL IgG (ϱ = –0.09 with platelets, p = 0.28; ϱ = –0.06 with hemoglobin, p = 0.47). **Panel B (HD patients):** aCL IgG shows a moderate negative correlation with platelet count (ϱ = –0.48, 95% CI: –0.62 to –0.31, p < 0.001) and a weaker negative correlation with hemoglobin (ϱ = –0.29, 95% CI: –0.47 to –0.09, p = 0.006). Platelets and hemoglobin are positively correlated in both groups. These stratified analyses confirm that the aCL–platelet association is specific to the HD population and not an artifact of pooling groups.

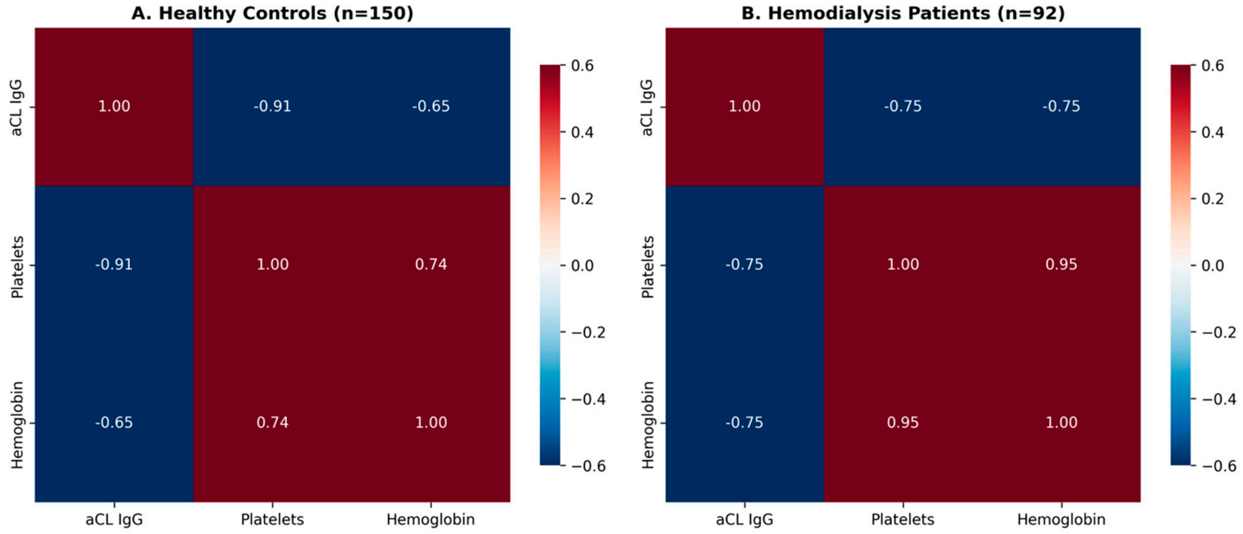

Supplement: Supplementary file 1 [file jcm-15-03269-s001.zip › jcm-4228758-supplementary.pdf]
